# USP42 drives nuclear speckle mRNA splicing via directing dynamic phase separation to promote tumorigenesis

**DOI:** 10.1038/s41418-021-00763-6

**Published:** 2021-03-17

**Authors:** Shuyan Liu, Taishu Wang, Yulin Shi, Lu Bai, Shanshan Wang, Dong Guo, Yang Zhang, Yangfan Qi, Chaoqun Chen, Jinrui Zhang, Yingqiu Zhang, Quentin Liu, Qingkai Yang, Yang Wang, Han Liu

**Affiliations:** The Second Affiliated Hospital, Institute of Cancer Stem Cell, Dalian Medical University, Dalian, Liaoning Province China

**Keywords:** Non-small-cell lung cancer, Deubiquitylating enzymes

## Abstract

Liquid–liquid phase separation is considered a generic approach to organize membrane-less compartments, enabling the dynamic regulation of phase-separated assemblies to be investigated and pivotal roles of protein posttranslational modifications to be demonstrated. By surveying the subcellular localizations of human deubiquitylases, USP42 was identified to form nuclear punctate structures that are associated with phase separation properties. Bioinformatic analysis demonstrated that the USP42 C-terminal sequence was intrinsically disordered, which was further experimentally confirmed to confer phase separation features. USP42 is distributed to SC35-positive nuclear speckles in a positively charged C-terminal residue- and enzymatic activity-dependent manner. Notably, USP42 directs the integration of the spliceosome component PLRG1 into nuclear speckles, and its depletion interferes with the conformation of SC35 foci. Functionally, USP42 downregulation deregulates multiple mRNA splicing events and leads to deterred cancer cell growth, which is consistent with the impact of PLRG1 repression. Finally, USP42 expression is strongly correlated with that of PLRG1 in non-small-cell lung cancer samples and predicts adverse prognosis in overall survival. As a deubiquitylase capable of dynamically guiding nuclear speckle phase separation and mRNA splicing, USP42 inhibition presents a novel anticancer strategy by targeting phase separation.

## Introduction

Intracellular compartmentalization is a notable method adopted by eukaryotic cells to spatially organize and coordinate diverse physiological functions. Such compartments form essential organelles that are enclosed by a single membrane, double membrane, or in some cases are membrane-less, such as the centrosome. Recent progress has revealed that liquid–liquid phase separation (LLPS) represents a universal approach to enable the formation of a wide range of membrane-less compartments [1–3]. These phase-separated structures, collectively called biomolecular condensates, are micron-scale, dynamic, multivalent interaction-driven assemblies widely distributed within eukaryotic cells, and their roles in various cellular events are beginning to be revealed from a phase separation perspective [4–6].

Protein posttranslational modifications play pivotal roles in the regulation of cellular physiology, and are involved in every biological process including signal transduction, transcriptional regulation, protein homoeostasis, and phase separation. A leading paradigm of phosphorylation-regulated phase separation has been convincingly demonstrated to potentiate signal transduction initiated from the T-cell receptor [7]. Ubiquitylation is another prevalent modification type, with counterbalancing E3 ligase and deubiquitylase (DUB) activities dynamically determining the amount of covalently attached ubiquitin entities residing on substrate proteins [8]. Interestingly, contradictory roles of ubiquitin molecules in regulating LLPS have been proposed. On the one hand, ubiquitin and K48-linked ubiquitin chains were shown to disrupt the LLPS of UBQLN2, a ubiquilin type adaptor, through noncovalent binding to enable substrate trafficking; on the other hand, K63-linked and linear ubiquitin chains were reported to induce LLPS of the autophagy receptor p62 to promote autophagic degradation [9, 10].

In addition to ubiquitin, emerging evidence has associated E3 ligase and DUB with LLPS. The speckle-type POZ protein, a substrate adaptor for the cullin-3 ring ubiquitin ligase complex, phase separates into nuclear speckles via oligomerization, representing an example of ubiquitin E3 ligase activity linked to LLPS compartments and cancer development [11, 12]. Furthermore, a *Drosophila* deubiquitylase called Otu was shown to undergo LLPS, which conferred enhanced enzymatic activity to extend the *Drosophila* lifespan [13]. Despite this intriguing evidence, the association of human DUBs with LLPS and ubiquitylation-mediated phase separation of DUB substrates has remained elusive until now. Recent advances have revealed two additional subfamilies of human deubiquitylases, motif interacting with Ub-containing novel DUB family (MINDY) and ZUP1, complementing the previously established ubiquitin-specific protease (USP), ovarian tumour protease (OTU), Jab1/Pad1/MPN-domain containing metalloenzyme (JAMM), ubiquitin C-terminal hydrolase (UCH), and Ataxin-3/Josephin-domain containing protein (Ataxin-3/Josephin) subclasses to expand human deubiquitylases to 7 subfamilies containing over 90 cysteine proteases (USP, OTU, UCH, Josephin, MINDY and ZUP1) and metalloenzymes (JAMM) [8, 14–16]. A subset of these isopeptidases has been revealed to distribute to specific subcellular locales, with their DUB activities implicated in corresponding cellular functions [17, 18].

In this study, we present evidence that human USP42 forms liquid droplets in the nucleus through LLPS. We confirm that the positively charged motifs in its C-terminal disordered region drive phase separation and nuclear speckle incorporation, with its DUB activity playing an essential role. Depletion of USP42 leads to a reduced number of nuclear speckles and attenuated cell growth. Mechanistically, USP42 governs the phase separation of the spliceosome component PLRG1 to regulate diverse mRNA splicing events, thus regulating gene expression involved in a wide range of cellular functions. As the enzymatic activity of USP42 can be potentially targeted by pharmacological intervention, further investigation on this deubiquitylase will likely shed light on future exploration of therapeutic strategies via targeting USP42-regulated phase separation.

## Materials and methods

### Cell lines

HeLa, U2OS, COS-7 and HEK293T cells obtained from the American Type Culture Collection (ATCC) were cultured in Dulbecco’s modified Eagle’s medium. H1299 cells acquired from ATCC were grown with RPMI 1640 medium (Gibco). Both media were supplemented with 10% foetal bovine serum (Gibco) and 1% penicillin/streptomycin (Thermo Fisher Scientific). Cell culture plates were obtained from Guangzhou Jet Bio-Filtration Co., Ltd. Cells were routinely maintained in a humidified atmosphere at 37 °C in a CO_2_ incubator (Thermo, 3111).

### Antibodies and other reagents

Rabbit anti-USP42 antibody for immunoblotting was purchased from Proteintech (18811-1-AP, Wuhan, China), and rabbit anti-USP42 antibody (PA5-66720) for immunofluorescence was purchased from Invitrogen. Antibodies against SC35 (S4045) and tubulin (T5168) were purchased from Sigma. Mouse anti-PLRG1 antibody (11914-1-AP) for immunoblotting was obtained from Proteintech, and rabbit anti-PLRG1 antibody (HPA035931) for immunofluorescence was obtained from Sigma. Anti-GFP antibody (ab6556) was purchased from Abcam. Mouse anti-V5 antibody (R96025) was from Invitrogen. Normal mouse IgG control for immunoprecipitation was from Santa Cruz Biotechnology. Secondary antibodies for immunofluorescence—goat anti-mouse and anti-rabbit IgG labelled with Alexa Fluor-594 or Alexa Fluor-488—were obtained from Invitrogen. Secondary antibodies for immunoblotting—Infra-red-labelled goat anti-mouse and anti-rabbit antibodies—were purchased from LI-COR.

### Plasmids and transfection

The DUB-expressing constructs were reported by Sowa et al. and were obtained from Addgene [19]. The coding sequences of human DUBs were shuttled into the pEGFP-C1 vector using standard molecular cloning to encode GFP-tagged fusion proteins. Similarly, truncated versions of USP42 were PCR amplified and inserted into pEGFP-C1 following restriction enzyme digestion. The catalytically inactive mutant (USP42-C120A) was generated via site-directed mutagenesis according to the manufacturer’s instructions (QuikChange, Agilent). USP42 truncations were also subcloned into the pET-28a backbone for His-tag purification. Plasmid sequences were verified by sequencing. Primer sequences are described in Table EV2. Plasmid transfection was carried out using Lipofectamine 3000 reagent (Invitrogen) according to the manufacturer’s instructions.

### Immunofluorescence

Immunofluorescence assays were performed as described previously [20]. In brief, treated cells were washed with PBS and fixed in 3% paraformaldehyde for 15 min at room temperature. Following incubation with ammonium chloride for 15 min, cells were permeabilized with 0.2% Triton X-100 to allow optional antibody labelling. Subsequently, the samples were blocked with 2% BSA for 30 min, and then incubated with primary antibody for 1 h at room temperature. After washing with PBS, the cells were stained with secondary antibody for 1 h at room temperature. Coverslips were mounted onto slides using Mowiol, and DAPI was added for nuclear visualization. Standard fluorescence images were captured using a fluorescence microscope (Olympus BX63, Japan). Confocal images were acquired with Leica laser scanning microscopes (TCS SP5 and SP8) and analyzed using the ImageJ programme. Super-resolution microscopy was performed using the 3D structured illumination-based DeltaVision OMX imaging system (GE).

### Fluorescence recovery after photobleaching (FRAP)

U2OS cells were seeded onto glass-bottom dishes and transfected with GFP-USP42 expressing constructs (GFP-USP42, GFP-USP42-C and GFP-USP42-C120A). GFP-labelled punctae were visualized by fluorescence microscopy and then bleached for 3 s using a 488 nm laser at an intensity of 60%. Post-photobleaching time-lapse images were captured and recovery fluorescence intensity was recorded at an interval of 2.5 s for a 180 s duration. The intensity of USP42 punctae from postbleaching images was normalized against that before photobleaching. The quantification data were analyzed and plotted using GraphPad Prism software (version 7).

### Immunoblotting

Cell lysates were prepared with the “RIPA” buffer (10 mM Tris-HCl pH 7.5, 150 mM NaCl, 1% Triton X-100, 0.1% SDS, and 1% sodium deoxycholate) supplemented with phenylmethylsulfonyl fluoride and Na_3_VO_4_ at 1 mM. Samples were subjected to centrifugation clearance before protein concentration determination with a BCA assay kit (Takara). Samples containing equal protein amounts were mixed with loading buffer and denatured for 5 min at 95 °C. After loading onto SDS-polyacrylamide gels, proteins were separated and then transferred to nitrocellulose membranes, which were blocked with 4% fat-free milk and incubated with primary antibodies at 4 °C overnight. Following PBS washes, the membrane was incubated with secondary antibodies for 1 h at room temperature. Finally, membranes were scanned on a LI-COR Odyssey imager, and protein band intensities were analyzed with the Image Studio programme (version 4.0).

### Lung cancer tissue specimens

Experimental procedures were approved by the Medical Ethics Committee of the Second Hospital of Dalian Medical University and signed consent forms were obtained. Surgically resected lung cancer and paracancerous normal tissue samples were collected at the Second Affiliated Hospital of Dalian Medical University. Freshly frozen tissues were sliced for protein and RNA extractions, with preparations analyzed by standard immunoblotting and RT-PCR assays.

### Generation of stable cells

To establish stable PLRG1 knockdown cell lines, pGIPZ vectors carrying PLRG1-specific shRNAs were obtained from Thermo Scientific, with the target sequences being sh1, TCGAATACAATAAGGTTAT and sh2, CGGTCATAATGCTATTATT. Lentivirus production was performed using HEK293T cells with the Open Biosystems TransLenti viral packaging system (Thermo Scientific) according to the manufacturer’s instructions. Packed viruses were used to infect H1299 cells, and positive clones were selected with puromycin at 2 μg/ml. Knockdown efficiency was validated through immunoblotting. To generate stable SS18-S-expressing H1299 cells, the SS18-S sequence was cloned into a pCDH vector that was cotransfected into HEK293T cells along with psPAX2 and pMD2.G packaging plasmids as described previously [21]. Harvested lentiviruses were used to treat H1299 cells, followed by puromycin (2 μg/ml) selection and subsequent verification of SS18-S expression by RT-PCR.

### CRISPR/Cas9-mediated gene ablation

A standard CRISPR/Cas9 protocol was followed to deplete USP42 in H1299 cells [22]. In brief, USP42-targeting guide RNA was designed using a web-based tool (http://crispr.mit.edu/), and the sequences were as follows: USP42-gRNA#1, GGCTTATTTTGCAAGGCGTG and USP42-gRNA#2, CTCGAATAAACTACAGCACC. Commercially synthesized oligonucleotides were inserted into the pSpCas9(BB)-2A-GFP (PX458) expression vector obtained from Addgene. Following validation by sequencing, the plasmid was transfected into H1299 cells. After 48 h, cells were harvested and processed for flow cytometry to select GFP-positive clones using a BD FACSAria II cell sorter. Single clones seeded into each well of a 96-well plate were cultured and expanded in an incubator before USP42 levels were examined by immunoblotting to confirm efficiency.

### Protein purification and in vitro phase separation assay

USP42-(742-1316) with or without GFP tag and GFP-PLRG1 sequences were transferred into the pET-28a plasmid to encode His-tagged proteins. Cloning was verified by sequencing, before plasmid transformation into the BL-21 (DE3) strain of *Escherichia coli*. Resultant bacteria were grown in LB media and protein expression was induced with 0.4 mM of IPTG at 37 °C for 12 h. His-tagged proteins were purified using Qiagen Ni-NTA agarose according to the manufacturer’s batch purification protocol and finally stored in buffer containing 25 mM HEPES pH 7.5 and 1 mM DTT. To detect in vitro phase separation of purified USP42 and PLRG1 proteins, stock preparation was diluted in phase separation buffer (12.5 mM HEPES pH 7.5 and 0.5 mM DTT) to reach the indicated concentrations, with or without crowding agents (PEG 400, 2000, and 4000) at 25% (w/v) and NaCl at concentrations ranging from 0 to 600 mM. Samples were finally examined by relief contrast (RC) and fluorescence microscopy (Olympus IX81, Japan).

### mRNA sequencing and alternative splicing (AS) analysis

Total RNA from parental H1299 cells and H1299 cells depleted of USP42 via the CRISPR/Cas9 approach was isolated using TRIzol reagent (Invitrogen) and subsequently cleaned with an RNAeasy Kit (Qiagen). The RNAs were digested in a column with RNase-free DNase according to the manufacturer’s instructions. Total RNA (less than 3 µg) was used to purify polyadenylated RNA using an Illumina TruSeq Total RNA Sample Prep kit according to the manufacturer’s instructions. We used the Ribo-Zero (Human) kit to remove rRNA. mRNA preparation was further analyzed using a Bioanalyzer (Agilent Technologies) prior to generation of a cDNA library with bar-coded ends. RNA-seq libraries were robotically prepared with an Illumina TruSeq Total RNA Sample Prep kit according to the manufacturer’s protocol. Cluster generation and sequencing were carried out by standard procedures on a HiSeq 2000 Illumina platform, and we used a paired-end sequencing protocol to generate reads at each end. The paired-end sequences were mapped to the human genome (hg18) using MapSplice 2.0.1.6 (default parameters) to discover splicing junctions. The mapped reads were further analyzed with Cufflinks to calculate the level of gene expression. The changes in splicing isoforms were analyzed using the MISO package with the annotation of all known AS events, and we filtered the results based on the PSI (percent spliced in) values.

### Splicing assay via semi-quantitative RT-PCR

Total RNA was isolated from transfected cells with TRIzol reagent (Invitrogen) according to the manufacturer’s instructions, followed by 1 h of DNase I (Invitrogen) treatment at 37 °C and subsequent heat inactivation. Total RNA (2 µg per condition) was then reverse-transcribed using SuperScript III reverse transcriptase (Invitrogen) with poly T primers, and one-tenth of the products were used as templates for PCR amplification (25 cycles). Primer sequences are described in Supplementary Table 2. RT-PCR products were separated on 2% agarose gels, and the amount of each splicing isoform was measured with ImageJ.

### Colony formation

The indicated cell line was seeded into either a 6-well plate (750 cells per well) or a 6-cm dish (2, 000 cells) and cultured in an incubator at 37 °C. Growth media were replenished every 48 h during a 2-week period. Cell colonies were fixed with methanol for 15 min. Following PBS washes, cells were stained with 0.1% crystal violet for 15 min. Images of cell colonies were captured using the Bio-Rad ChemiDoc XRS+ system and quantified with the ImageJ programme.

### Statistics

Experiments were carried out with three biologically independent repeats. Data are illustrated as the mean ± SEM (standard error of the mean). Statistical differences were generally evaluated by performing Student’s *t*-test (two tailed) in GraphPad Prism software (version 7). Statistical analyses of sequencing data were conducted in R 3.6.2 software. *P* < 0.05 presents a significant difference.

## Results

### Systematic survey reveals USP42 as a phase-separated DUB in the nucleus

**Fig. 1:**
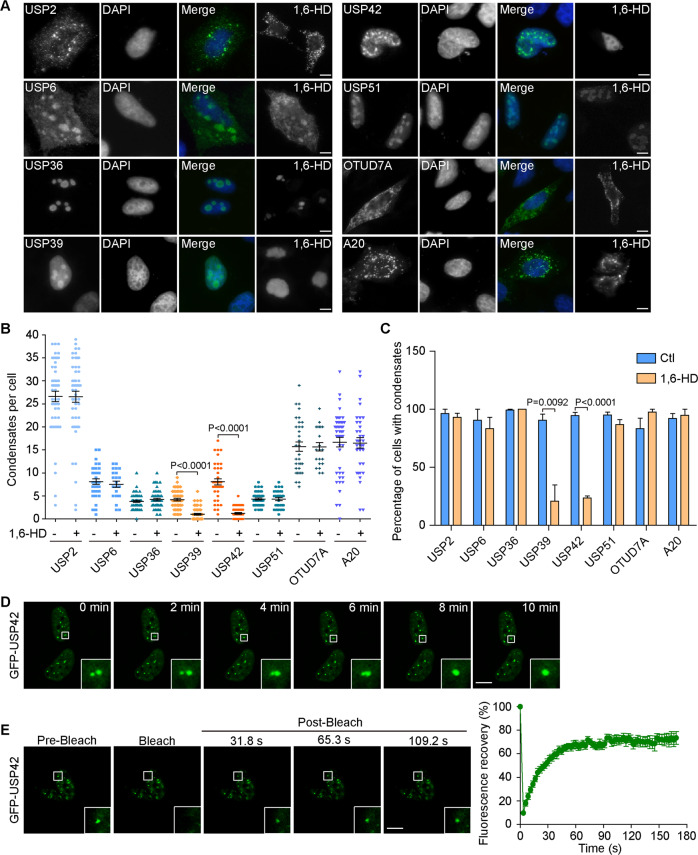
Systematic survey identifies USP42 as a phase-separated DUB in the nucleus. **A** Representative images showing subcellular localizations of indicated DUBs through transient expression in HeLa cells under steady state conditions or with 1,6-hexanediol (1,6-HD) treatment (3%, 7 min). Nucleus was stained with DAPI. Scale bar = 10 μm. **B** Quantification data of condensates formed per cell with or without 1,6-HD treatment as in (**A**) from three independent experiments. **C** Quantification of cell percentages with condensate formation from three independent experiments. **D** Time-lapse images captured by confocal microscopy with magnified insets showing the fusion of two adjacent GFP-USP42 punctae in U2OS cells within a 10-min duration. Scale bar = 10 μm. **E** Representative time-lapse FRAP images acquired in U2OS cells with magnified insets showing the pre-bleach and recovery signals of GFP-USP42. The curve on the right shows the relative fluorescence intensity quantified during FRAP recovery (*n* = 3). Scale bar = 10 μm.

Biomolecular condensates formed through LLPS maintain locally elevated concentrations of resident proteins, which frequently show intracellular punctate structures that allow sensitive detection through imaging methodologies [2, 3, 23]. To interrogate the associations of human DUBs with intracellular biomolecular condensates, we systematically surveyed the subcellular distribution of 71 human deubiquitylases encompassing 6 DUB subfamilies [8, 17, 24, 25]. Using a GFP or Flag tagging approach to optimize fluorescence signals, we were capable of visualizing their intracellular localizations with transient transfection in HeLa cells (Supplementary Figs. 1 and 2, Supplementary Table 1). Among this panel, 8 candidates, USP2, USP6, USP36, USP39, USP42, USP51, OTUD7A, and A20, consistently showed punctate structures in fluorescence microscopic analyses (Fig. 1A). In particular, USP36, USP39, and USP51 exhibited nucleolus-like staining, while USP2, USP6, OTUD7A, and A20 showed cytoplasmic condensates but USP42 displayed nuclear speckle-like staining. Taking advantage of the development of 1, 6-hexanediol as a convenient tool to disrupt LLPS, we examined the morphological changes of these condensates with hexanediol treatment [26, 27]. Representative images from Fig. 1A demonstrate that 1, 6-hexanediol readily dissolved punctae formed by GFP-tagged USP39 and USP42 but left the others unaffected, and quantification data of condensates formed by these DUBs indicate the phase separation potential of these two candidate DUBs (Fig. 1B, C).

Considering its unique nuclear puncta localization, we focused on USP42 for further investigation. We first corroborated its subcellular distribution by transiently expressing GFP-USP42 in U2OS and COS-7 cells. Consistent with the data obtained in HeLa cells, GFP-USP42 formed similar nuclear punctae in these cells (Supplementary Fig. 3A. B). We then moved to use U2OS cells in further fluorescence studies to improve image quality. To verify whether USP42 forms phase-separated liquid droplets in the nucleus, we conducted confocal microscopy and FRAP analyses to investigate the fluidity of GFP-USP42 punctae. Intriguingly, in live cell assays, GFP-USP42 punctae demonstrated fluid features through fusion phenomena; USP42 droplets also showed effective FRAP recovery within minutes after photobleaching (Fig. 1D, E). Collectively, these observations infer USP42 is a phase-separated DUB in the nucleus.

### USP42 C-terminal disordered region confers phase separation features

**Fig. 2:**
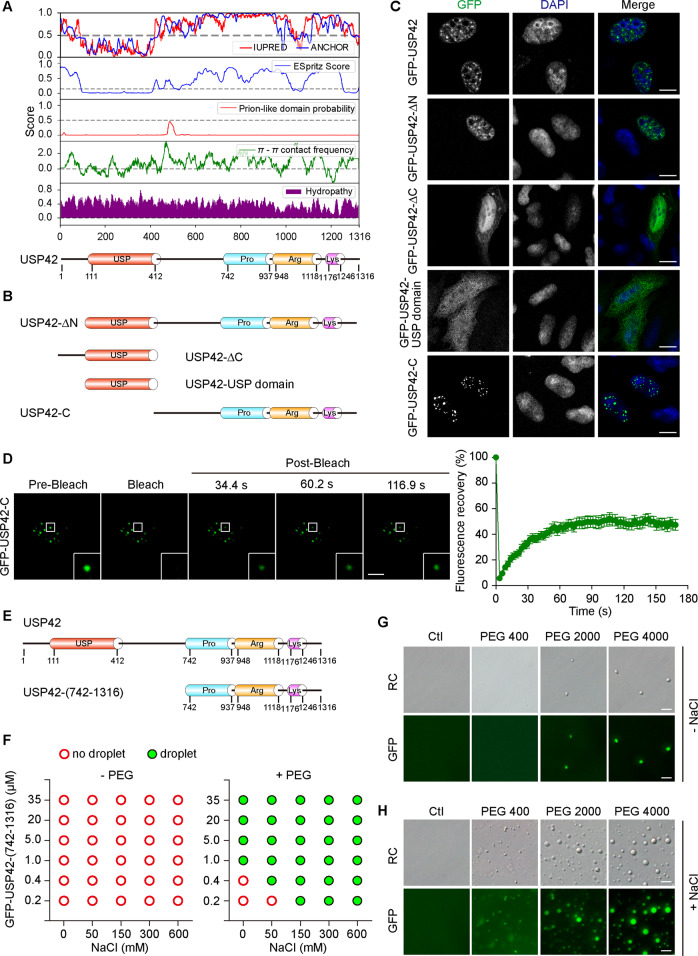
USP42 C-terminal disordered region confers phase separation features. **A** Analysis of USP42 amino acid sequence to assess intrinsic disorder tendency (IUPRED and ESpritz), prion-like domain, Pi–Pi contact, and hydropathy as indicated. Below diagram shows USP42 domain structure at corresponding residues. **B** Schematic illustration demonstrates engineered USP42 expression constructs as indicated. **C** The distribution of GFP-tagged USP42 variants detected in U2OS cells via transient transfection and fluorescence assays. Nucleus was stained with DAPI. Scale bar = 10 μm. **D** Representative time-lapse images with magnified insets showing FRAP recovery of puncta formed by GFP-USP42-C variant. The curve on the right shows the relative quantification of fluorescence signal recorded pre- and post-bleach (*n* = 3). Scale bar = 10 μm. **E** Domain architectures of USP42 full length and USP42-(742-1316) truncation. **F** Summarized diagram showing the in vitro phase separation of purified GFP-USP42 fusion proteins in the absence (left) or presence (right) of the crowding agent PEG 4000 (25%). A range of concentrations for both USP42 and NaCl were included as indicated. Formation of phase separation was assessed by droplet observation under the microscope. **G** Representative relief contrast (RC) and fluorescence images showing in vitro condensate formation of purified GFP-USP42 truncation with or without crowding agents (PEG 400, 2000, and 4000) as indicated. Scale bar = 5 μm. **H** Representative RC and fluorescence images demonstrating enhanced condensate formation of purified GFP-USP42 truncation in the presence of NaCl. Scale bar = 5 μm. All experiments were performed with three biological repeats.

To gain further insights into the physical and chemical features of the USP42 amino acid sequence, we analyzed its disordered regions, prion-like composition, Pi–Pi contact frequency, and hydrophobicity features using the integrated PhaSepDB database (http://db.phasep.pro/) [28–30]. Amino acid sequence analysis revealed that, apart from its USP domain, the USP42 protein sequence was predicted to be intrinsically disordered by IUPred2A and ESpritz (Fig. 2A) [31, 32]. Intrinsically disordered regions (IDRs) have been closely implicated in phase separation [33–35]. To dissect the contribution of its predicted disordered region and USP domain to the phase separation features of USP42, we generated a series of truncated versions of GFP-USP42 (Fig. 2B). With these constructs, we carried out confocal microscopy to examine their subcellular distribution. In keeping with sequence analysis, the disordered region that is C-terminal to the USP domain appeared to dominate the subcellular localization of USP42, as two constructs lacking this region (USP42-∆C and USP42-USP domain) exhibited diffuse distribution throughout the cell, while this region alone displayed nuclear puncta localization (Fig. 2C; Supplementary Fig. 3C).

We then sought to examine whether the predicted disordered region of USP42 itself could show phase separation features. To this end, we expressed GFP-USP42-C in U2OS cells and performed FRAP assays. As illustrated in Fig. 2D, punctae formed by this truncation showed effective FRAP, although to a lesser extent compared to full-length—recovery to ~40% in truncation versus above 70% in wild type. Next, we investigated the in vitro phase separation of the USP42 disordered region using purified proteins. Owing to the difficulties in the purification of GFP-USP42 (170 KD) and GFP-USP42-C (126.5 KD), we further truncated USP42 to generate the GFP-USP42-(742–1316) (91.6 KD) variant, which contained proline-rich, arginine-rich, and lysine-rich domains (Fig. 2E). Using a His-tag-mediated approach, we achieved satisfactory purity and yield for subsequent in vitro analyses. Although no liquid droplet was observed with an escalation concentration up to 35 µM for purified USP42 truncation, we readily detected condensate formation in the presence of the crowding agent polyethylene glycol (PEG 400, 2000, and 4000) through both RC and fluorescence microscopy observations that presented consistent overlay, inferring the phase separation property of the USP42 C-terminal region (Fig. 2F–H). Interestingly, in the presence of PEG, our observations also revealed that the addition of NaCl gave rise to substantially enhanced USP42 phase separation that saturated at near physiological concentrations (150 mM) (Fig. 2F–H; Supplementary Fig. 3D). Furthermore, the N-terminal truncation of USP42 (1–412) that lacked an IDR sequence was unable to phase separate but formed aggregates in vitro, while a longer truncation construct that included an IDR sequence restored phase separation capabilities (Supplementary Fig. 3E). Collectively, it appears that the in vitro condensate formation of USP42 prefers an environment with molecular crowding and physiological salt concentrations, which likely mimicks the physiological milieu to facilitate phase separation.

### USP42 phase separates into SC35-positive nuclear speckles

**Fig. 3:**
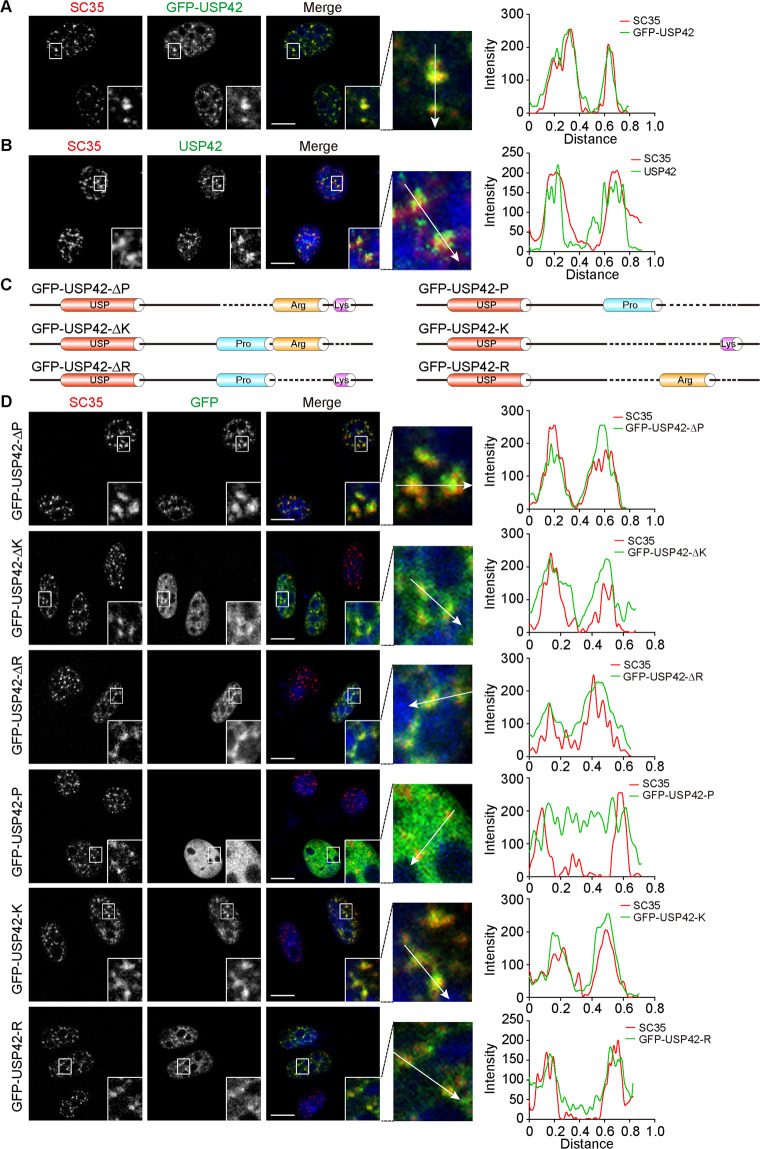
Efficient nuclear speckle incorporation of USP42 relies on positively charged residues. **A** Representative confocal micrographs with magnified insets showing colocalization of GFP-USP42 with endogenous SC35 detected in U2OS cells transiently expressing GFP-USP42. Graph on the right shows quantification. DAPI stains the nucleus. Scale bar = 10 μm. **B** Representative confocal sections with magnified insets illustrating colocalization of endogenous USP42 with SC35 in HeLa cells. Graph on the right shows quantification. Nucleus was stained with DAPI. Scale bar = 10 μm. **C** Schematic diagram of USP42 constructs with removal of one or two of the three motifs locating at USP42 C-terminus (proline-, arginine- and lysine-rich domains as indicated). GFP was tagged at N-terminus but omitted in the drawing to save space. **D** Immunofluorescence assays were conducted to inspect the colocalization of GFP-tagged USP42 mutants with endogenous SC35. Representative confocal micrographs were shown with magnified insets. Graphs on the right show quantification. Nucleus was stained with DAPI. Scale bar = 10 μm. All experiments were performed with three biological repeats.

Having proven that USP42 is a phase-separated DUB, we moved to study its physiological relevance. Based on the characteristics of its nuclear distribution, we speculate that USP42 could possibly localize to nuclear speckles, which are recognized as biomolecular condensates [3, 36]. Nuclear speckles have been linked to multiple splicing-associated functions and revealed to harbour many splicing factors including serine/arginine-rich splicing factor 2 (more commonly known as SC35), which is a resident nuclear speckle protein essential for pre-mRNA splicing [37, 38]. To this end, we carried out confocal microscopy assays using the well-established marker SC35 [39, 40] and observed evident colocalization of GFP-USP42 with SC35-positive nuclear speckles (Fig. 3A). Moreover, the nuclear speckle distribution of USP42 was further substantiated with the detected colocalization of endogenous USP42 and SC35 (Fig. 3B; Supplementary Fig. 4A). Considering the competency of the USP42 C-terminus in nuclear puncta formation, we sought to pinpoint the exact sequence motif governing nuclear localization as well as the corresponding underlying mechanisms. Hence, we assessed the involvement of the proline-, arginine-, and lysine-rich motifs in the nuclear speckle distribution of USP42 using a panel of constructs with deletions in any one or two of the 3 motifs (Fig. 3C). Confocal microscopy data showed that the removal of any single motif refrained from disturbing colocalizations with SC35 (Fig. 3D). However, retaining either the arginine- or lysine-rich domain alone but not the proline-rich motif effectively maintained punctate structures of GFP-USP42 as well as nuclear speckle localization (Fig. 3D; Supplementary Fig. 4B, C). Taken together, the results from localization assays with mutated USP42 proteins suggest that the nuclear speckle distribution of USP42 is likely dependent upon positive charges embedded in its arginine- and lysine-rich domains. Given that nuclear speckles harbour RNAs that are negatively charged, we examined GFP-USP42 distribution in cells treated with RNase [41, 42]. Nevertheless, following RNA depletion, GFP-USP42 punctae appeared to be more condensed and showed even enhanced colocalization with SC35, suggesting that the integration of USP42 into nuclear speckles was likely mediated through protein–protein interactions rather than protein-RNA interactions (Supplementary Fig. 4D).

**Fig. 4:**
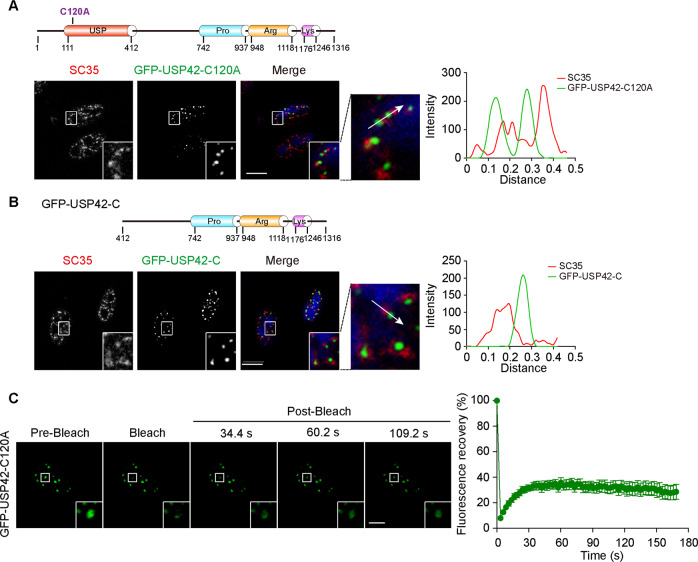
USP42 DUB activity is required for its efficient nuclear speckle localization and phase separation. **A**, **B** U2OS cells transfected with GFP-USP42-C120A (catalytically inactive) and GFP-USP42-C truncation expression constructs (domain structures as indicated without GFP tag) were analyzed by immunofluorescence to inspect their colocalization with endogenous SC35. Representative confocal sections were shown with magnified insets. Graphs on the right show quantification. Nucleus was stained with DAPI. Scale bar = 10 μm. **C** FRAP analysis of the droplets formed by GFP-USP42-C120A in U2OS cells, with relative fluorescence intensities shown in the curve on the right (*n* = 3). Scale bar = 10 μm.

Subsequently, we investigated the implications of DUB activity in LLPS and the nuclear speckle distribution of USP42 by expressing catalytically inactive mutant of GFP-USP42 (C120A) in U2OS cells. Figure 4A shows that USP42-C120A also forms nuclear punctate structures. However, unlike wild-type punctae, punctae formed by USP42-C120A showed no overlap with SC35 foci but were located adjacent to each other. These observations with USP42-C120A are consistent with those made for the USP42 C-terminus, which lacks the USP domain (Fig. 4B). Therefore, it seems that USP42 DUB activity is obligatory for its effective incorporation into nuclear speckles. Given that USP42-C120A still maintains an intact C-terminus that harbours a disordered region, we wondered whether this catalytically inactive mutant still displays fluid characteristics. Hence, we conducted confocal microscopy and FRAP analyses using live cells. Interestingly, although the USP42-C120A condensates failed to integrate into neighbouring nuclear speckles, these structures nevertheless exhibited fluid features and showed FRAP recovery, although to a lesser extent compared to wild type but resembled how the C-terminus truncation performed (Figs. 4C and 2D). Taken together, our findings suggest that the DUB activity-driven nuclear speckle incorporation of USP42 likely enhances its dynamic fluidity.

### USP42 controls PLRG1 phase separation and integration into nuclear speckles

**Fig. 5:**
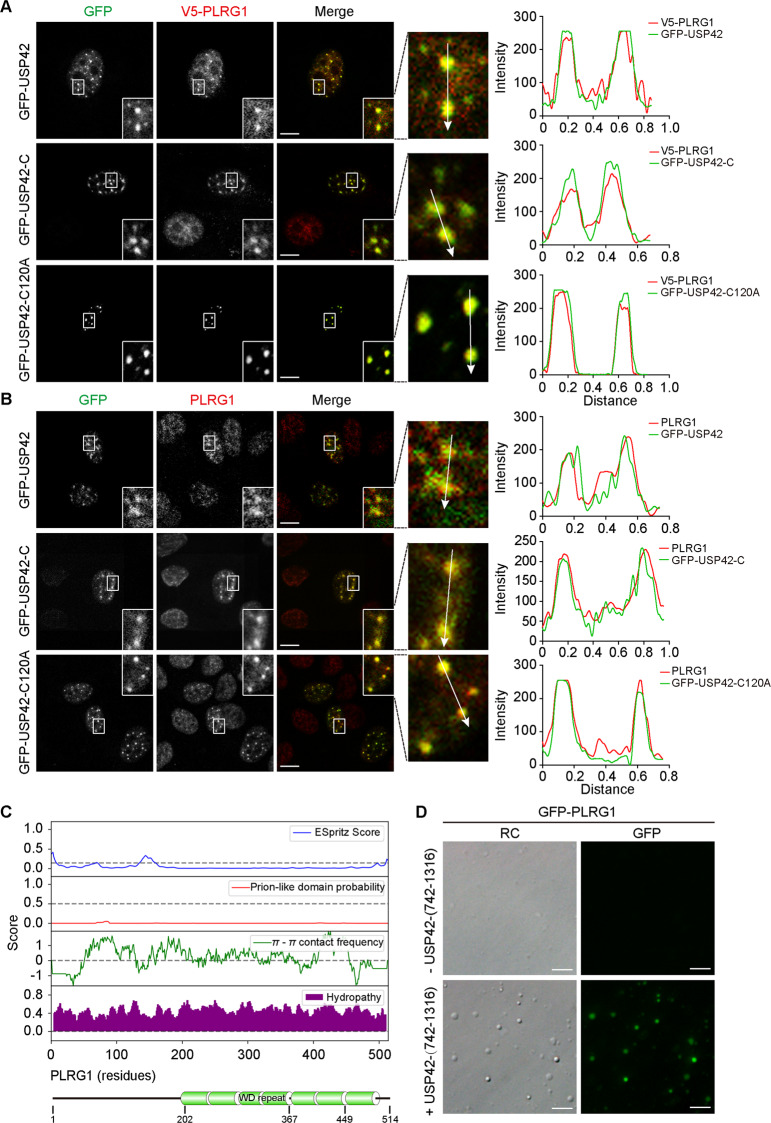
USP42 associates with and drives the phase separation of PLRG1. **A** U2OS cells were transfected with constructs expressing GFP-tagged USP42 proteins (wild-type, C-terminus, and catalytically inactive C120A) together with V5-tagged PLRG1 plasmid to allow immunofluorescence analysis of subcellular distribution of exogenous proteins as indicated. Representative confocal micrographs with magnified insets were presented to illustrate colocalizations. Graphs on the right show quantification. Scale bar = 10 μm. **B** Immunofluorescence analysis of exogenous GFP-tagged USP42 variants and endogenous PLRG1 in U2OS cells. Representative confocal images were shown with magnified insets to demonstrate colocalization. Relative to wild-type USP42, the two versions devoid of DUB activity (C-terminus and C120A) effectively concentrated endogenous PLRG1 to form more intensely stained assemblages. Graphs on the right show quantification. Scale bar = 10 μm. **C** Bioinformatics analysis of PLRG1 amino acid sequence to examine disorder tendency, prion-like domain, Pi–Pi contact, and hydropathy using the web-based phase separation database PhaSepDB. Below diagram shows PLRG1 domain structure at corresponding residues. **D** Representative relief contrast (RC) and fluorescence images showing in vitro phase separation of purified GFP-tagged PLRG1 (0.25 μM) with or without non-GFP-tagged USP42 (15 µM) proteins. PEG 4000 (25%) and NaCl (150 mM) were included in all assays. Scale bar = 5 μm. All experiments were performed with three biological repeats.

As part of our efforts to identify potential substrates for USP42, we interrogated the human DUB interactome database using the Harper Lab Proteomics Website (http://besra.hms.harvard.edu/ipmsmsdbs/comppass.html) for high-confidence candidate interacting proteins (HCIPs) [19]. In light of the subcellular distribution of USP42, we searched its HCIPs for nuclear speckle-associated proteins and identified pleiotropic regulator 1 (PLRG1) as our top candidate [43]. We successfully confirmed their interaction via performing coimmunoprecipitation assays, with results showing evident association of PLRG1 with both wild-type and the C-terminus of USP42 (Supplementary Figure 5A). Subsequently, we exogenously expressed GFP-USP42 proteins (wild-type, C-terminus, and C120A) together with V5-tagged PLRG1 in U2OS cells and investigated their distributions. Consistent with the coimmunoprecipitation data, all three versions exhibited evident colocalization with PLRG1, suggesting that the USP42 C-terminus drives the interaction with PLRG1 and that its enzymatic activity is dispensable (Fig. 5A). Congruently, in subsequent immunofluorescence analyses with exogenous expression of USP42 wild type, C-terminus, and C120A to examine endogenous PLRG1 distribution, the latter two variants that lacked enzymatic activity led to markedly more intense PLRG1 foci compared to those in wild-type-transfected and untransfected cells, thus giving rise to enhanced colocalizations (Fig. 5B). These findings collectively indicate that the USP42 C-terminus is capable of efficiently conscripting endogenous PLRG1.

Bioinformatic analysis of the PLRG1 sequence nonetheless revealed no evident disordered region or prion-like domain that is linked to phase separation potential (Fig. 5C). Furthermore, no condensation was detected in vitro with purified PLRG1 proteins, thus indicating limited capability to phase separate on its own. However, when the USP42 C-terminal fragment (non-GFP-tagged) was added, GFP-tagged PLRG1 was readily incorporated into liquid droplets as revealed by RC and fluorescence microscopy (Fig. 5D). Therefore, evidence gathered from both in vivo and in vitro assays appears to comply with the “scaffold” and “client” models of biomolecular condensate composition for USP42 and PLRG1, respectively [3].

### USP42 depletion hinders cell proliferation and alters nuclear speckle morphology

**Fig. 6:**
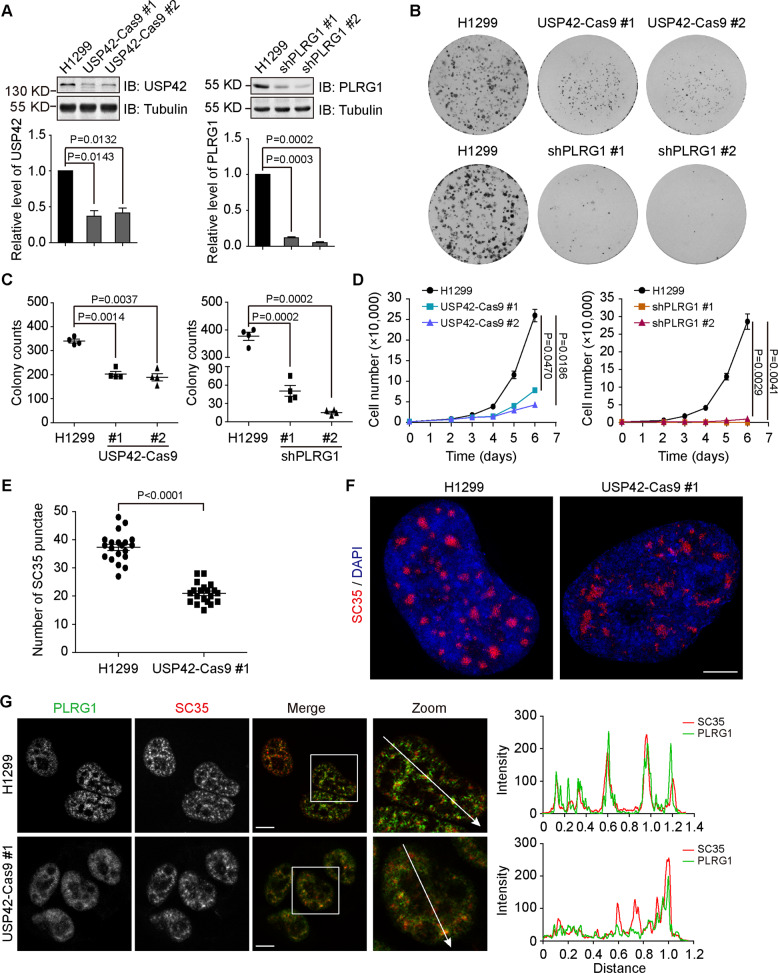
USP42 depletion influences cell growth, nuclear speckles, and PLRG1 distribution. **A** The efficiency of USP42 and PLRG1 depletion was determined by immunoblotting in indicated stable H1299 cell lines. Tubulin blots confirm equal loading. Below column charts show quantification data of band intensities (*n* = 3). *P* values were calculated by paired *t*-test. Error bars represent standard error of the mean. **B** Colony formation assays of parental H1299 cells and stable clones with USP42 and PLRG1 depletion as indicated. **C** Quantification data show colony numbers from indicated cell lines. *P* values were calculated by performing two-tailed Student’s *t*-test. Error bars represent standard error of the mean, *n* = 4. **D** Cell proliferation assays of control and stable H1299 cells with USP42 depletion (USP42-Cas9 #1 and USP42-Cas9 #2) and PLRG1 knockdown (shPLRG1 #1 and shPLRG1 #2). Cell numbers over a 6-day incubation period were recorded and plotted (*n* = 3). Error bars represent the standard error of the mean, with *P* value calculated by Student’s *t*-test. **E** The numbers of SC35 foci in parental and USP42-depleted H1299 cells were quantified and plotted (*n* = 3). *P* value was calculated by performing two-tailed Student’s *t* test. Error bars represent standard error of the mean. **F** Super-resolution microscopy (SIM) images showing the morphology of nuclear speckles marked with SC35 in parental and USP42-depleted H1299 cells. Nucleus was stained with DAPI. Scale bar = 5 μm. **G** Immunofluorescence analysis of endogenous PLRG1 and SC35 in H1299 cells with or without USP42 depletion. Representative confocal micrographs are shown with magnified insets to illustrate colocalization in two cell lines. Graphs on the right show quantification. Scale bar = 10 μm.

After comparing different methodologies to deplete USP42 expression, we achieved the best efficiency using CRISPR/Cas9 although without complete knockout, instead obtaining reduction to ~50%, likely due to one allele disruption (Fig. 6A). Since USP42 has been reported to regulate the stability of p53, which affects multiple facets of cellular physiology, we selected H1299 cells with a p53-null background to characterize the influence of USP42 depletion [44]. Phenotypically, the depletion of either USP42 or PLRG1 resulted in attenuated cell growth, as revealed by colony formation and cell proliferation assays (Fig. 6B–D). Interestingly, a decrease in USP42 expression led to a significantly reduced number of nuclear speckles marked with SC35 (Fig. 6E; Supplementary Fig. 5B). When examined through super-resolution microscopy, SC35 foci in USP42-depleted cells tended to form even more irregular shapes and were not as compact as those in parental cells (Fig. 6F). In addition, the distribution of PLRG1 appeared to be more diffuse in USP42-depleted cells than in parental H1299 cells, and its colocalization with SC35 was attenuated in cells lacking USP42 (Fig. 6G). Nevertheless, USP42 downregulation did not lead to significant changes in the expression levels of PLRG1 (Supplementary Fig.  5C). In addition, SC35 and USP42 condensates were not significantly affected by PLRG1 depletion (Supplementary Fig. 5D). Furthermore, we investigated the influence of ubiquitin overexpression on USP42 condensate formation and fluidity, with the results from immunofluorescence and FRAP analyses showing no considerable changes in cells with ubiquitin overexpression (Supplementary Fig. 6A–F).

### USP42 influences diverse mRNA splicing events and is associated with the prognosis of non-small-cell lung cancer

**Fig. 7:**
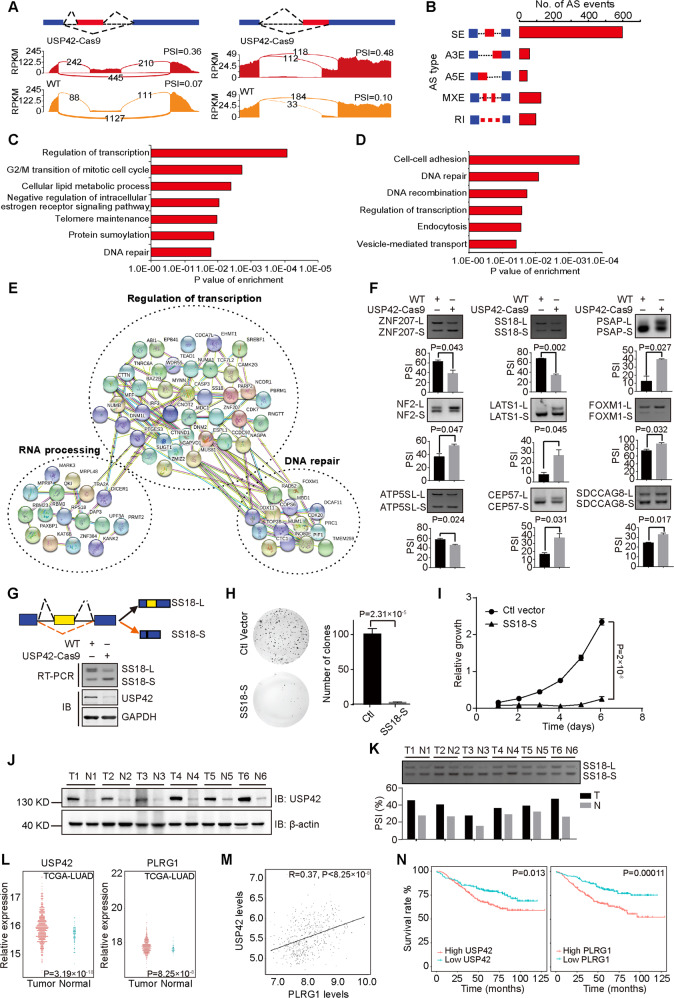
USP42 modulates alternative splicing of cancer-related genes through regulating PLRG1. **A** Examples of alternative exon and alternative use of 3′ ss affected by USP42. Numbers of exon-junction reads and PSI were indicated. **B** Quantification of different AS events affected by USP42. **C** Gene-ontology of USP42-regulated AS targets. Fisher exact *P* values were plotted. **D** Gene ontology of AS targets regulated by both USP42 and PLRG1. Fisher exact *P* values were plotted. **E** Functional association network of USP42- and PLRG1-regulated AS targets. **F** Validation of candidate AS events by semi-quantitative RT-PCR using parental and USP42-Cas9 H1299 cells. The PSI means from three independent experiments were plotted (*P* values obtained from paired *t*-test). Error bars represent standard error of the mean. **G** The splicing change of SS18 was measured with parental and USP42-Cas9 H1299 cells. The protein levels of USP42 were examined by immunoblotting. GAPDH was also probed as loading control. **H** The proliferation of H1299 cells stably transfected with SS18-S-expressing or control vector was determined by colony formation assays. All experiments were performed with three biological repeats, with quantified relative colony numbers plotted. *P* value was calculated by Student’s *t* test. Error bars represent standard error of the mean. **I** H1299 cells stably expressing SS18-S or control were grown for 6 days, with cell numbers measured every day. The changes of cell numbers were compared to day 0. The means from three replicates were plotted and *P* value was calculated by Student’s *t* test. Error bars represent standard error of the mean. **J** USP42 levels from six pairs of NSCLC (T) and adjacent normal (N) tissues were analyzed by immunoblotting. β-actin blot shows equal loading. **K** Total RNAs were isolated from paired NSCLC tumours and normal tissues. Splicing of SS18 was assayed by RT-PCR, with below column chart showing PSI values. **L** The mRNA levels of USP42 and PLRG1 in lung adenocarcinoma patients as reported from TCGA were analyzed and plotted. *P* values were calculated with unpaired *t*-test. **M** linear regression analysis of the relative levels of USP42 and PLRG1 in GSE11969 and GSE31210 datasets was shown. *R* = 0.37, *P* < 8.25 × 10^−8^. **N** The groups of lung cancer patients with distinct USP42 or PLRG1 levels were obtained from GSE11969 and GSE31210 datasets to analyze the overall survival (P values were calculated by log-rank test).

Since PLRG1 is a component of the spliceosome that is involved in pre-mRNA splicing, we next investigated whether USP42 could influence AS by regulating PLRG1 [45, 46]. RNA-seq analyses of parental H1299 cells and those depleted of USP42 identified a wide array of USP42-regulated AS events with an obvious change in the percent-spliced-in (PSI) value (≥0.15). Figure 7A demonstrates the read tracks of two examples. Various types of AS are regulated by USP42 and PLRG1, including skipped exons, alternative 5′ ss exons (A5Es), alternative 3′ ss exons (A3Es), retained introns, and mutually exclusive exons (Fig. 7B; Supplementary Fig. 7A). We further analyzed the cellular functions of USP42- and PLRG1-regulated AS events using gene ontology and found genes implicated in multiple pathways, including the regulation of transcription, G2/M transition, and DNA repair (Fig. 7C; Supplementary Fig. 7B). Importantly, the key cellular functions of USP42-regulated AS events overlapped with those of PLRG1-regulated AS events, and subsequent analysis of the common AS events regulated by USP42 and PLRG1 revealed involvement in functions such as the regulation of transcription and DNA repair (Fig. 7D; Supplementary Fig. 7C). Interestingly, many of the commonly regulated splicing targets are functionally connected into well-linked interaction networks, as judged by STRING (Search Tool for the Retrieval of Interacting Genes/Proteins), which contain genes regulating transcription, DNA repair and RNA processing (Fig. 7E).

Subsequent validation of splicing changes in nine newly identified targets that were arbitrarily chosen to include cancer-associated genes confirmed the impact of USP42 regulation on endogenous AS events (Fig. 7F). To interrogate the outcome of such splicing regulation, we focused on SS18, which produces two splicing isoforms. The *SS18* gene encodes the Synovial Sarcoma Translocated To X Chromosome Protein (SSXT), which is also called the SS18 Subunit Of BAF Chromatin Remodelling Complex and has been implicated in different nuclear functions, including transcriptional coactivation and chromatin regulation [47, 48]. Depletion of USP42 appeared to shift SS18-L to SS18-S (Fig. 7G). Importantly, the expression of SS18-S was linked to dramatically inhibited cell growth, as revealed by colony formation and cell proliferation assays (Fig. 7H, I; Supplementary Fig. 7D). Taken together, these results indicate potential roles of USP42 in cancer progression by regulating AS of cancer-related genes.

The protein levels of USP42 in surgically collected lung cancer and adjacent normal tissue pairs from six patients were assessed, and the results showed elevated expression of USP42 in cancer specimens relative to normal tissues (Fig. 7J). Consistently, the splicing of SS18 was shifted in tumour samples, with SS18-L being the predominant isoform (Fig. 7K). In addition, we analyzed USP42 and PLRG1 expression in the TCGA dataset and found significant upregulation of USP42 and PLRG1 in lung adenocarcinoma and squamous carcinoma (Fig. 7L; Supplementary Fig. 7E). Further linear regression analysis revealed a positive correlation between USP42 and PLRG1 expression (*R* = 0.37, *P* < 8.25 × 10^−8^) (Fig. 7M). To explore the clinical relevance of USP42 and PLRG1 expression in lung cancer, we analyzed the overall survival of cancer patients using datasets from GSE11969 and GSE31210. Strikingly, increased expression of USP42 or PLRG1 negatively correlated with patient survival, thus indicating a tumour-promoting role for both USP42 and PLRG1 in human lung cancer (Fig. 7N).

## Discussion

Biomolecular condensates formed through LLPS have emerged as widely distributed organizers in eukaryotic cells that enable spatiotemporal control of biochemical reactions with implications in various cellular events that are beginning to be elucidated from a phase separation perspective [1, 2, 49]. Despite the identification of an increasing number of membrane-less compartments driven by LLPS and insights into the molecular grammar underlying the assembly of condensates, our understanding of the dynamic regulation of the composition and functionality of biomolecular condensates has remained limited until now [3, 50]. Intriguingly, protein phosphorylation has provided some leading examples to illustrate the regulation of protein coalescence through phosphorylation-mediated phase separation [7, 51]. Ubiquitylation, another prevailing type of posttranslational modification, has also been implicated in regulating the composition of phase-separated structures [9, 10].

**Fig. 8:**
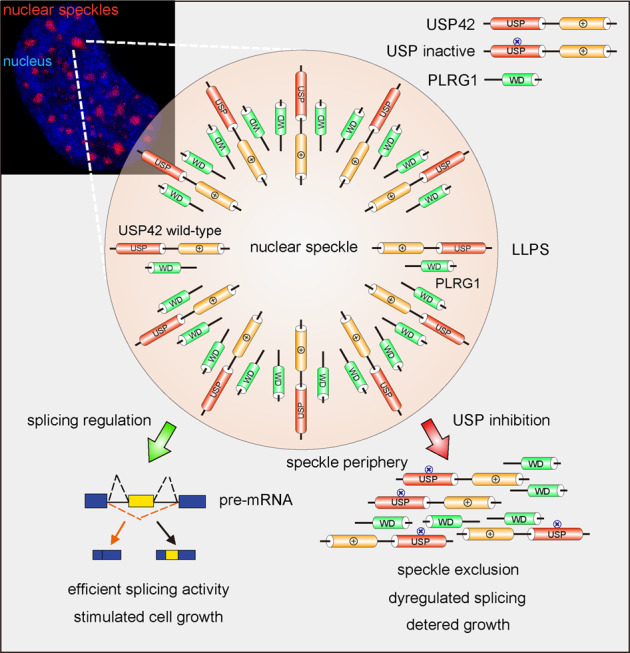
A working model depicting PLRG1-mediated alternative splicing driven by USP42 phase separation. The intrinsically disordered regions mediate the phase separation of USP42 in a positive charge-dependent manner. USP42 DUB activity dominates its efficient incorporation into nuclear speckles to allow enhanced motility. On the contrary, inactivation of the USP domain renders speckle exclusion of USP42, leaving phase-separated USP42 located to speckle periphery with limited motility. However, USP42 is competent to recruit PLRG1 both within and beside nuclear speckles, with nuclear speckle incorporation of PLRG1 conferring efficient splicing activity that is potentially exploited during tumorigenesis to enable stimulated cell growth.

In this study, we conducted a systematic survey to examine the association of human DUBs with phase separation characteristics and present evidence showing that USP42 forms liquid droplets in the nucleus through LLPS. Further investigation revealed that USP42 contains IDRs and phase separates into nuclear speckles via a positive charge-mediated mechanism (Fig. 8). Despite the existence of various RNAs in nuclear speckles, the incorporation of USP42 is nevertheless observed to be mediated through protein–protein interactions. Although the USP42 C-terminal sequence per se is competent to form interactions that enable its phase separation, endogenous USP42 likely also associates with negatively charged moieties within nuclear speckles to allow improved motility and substrate encounters. Importantly, USP42 deubiquitylase activity is revealed to be indispensable for its effective nuclear speckle incorporation, which can potentially be exploited to develop therapeutic strategies through pharmacologically targeting its enzymatic activity. This intriguing aspect warrants further investigation on USP42, considering the challenges that we face to interfere with phase separation owing to the lack of feasible tools for now [52].

USP42 governs the phase separation of the spliceosome component PLRG1 to regulate a range of mRNA splicing events that are linked to diverse cellular functions and the clinical prognosis of lung cancer patients. Hence, our observations also link potential RNA splicing activities to nuclear speckles. These enigmatic nuclear condensates were initially recognized as storage sites for factors required for transcription and RNA processing, while continuing advances have associated these structures with further active functionality to boost gene expression and facilitate RNA processing [41, 53–55]. Interestingly, USP42 was also proposed to regulate transcription by deubiquitylating histone H2B [56]. Hence, nuclear speckle-localized USP42 is likely implicated in multiple steps of RNA biogenesis, which warrants further investigation. As a cysteine protease, USP42 holds the potential to serve as a therapeutic target in cancer treatment [57, 58]. With its DUB activity providing an accessible handle to interfere with the phase separation of nuclear speckle components, USP42 could represent a leading paradigm to shed light on the development of LLPS-targeted therapies.

## Supplementary information

Supplementary Figure 1

Supplementary Figure 2

Supplementary Figure 3

Supplementary Figure 4

Supplementary Figure 5

Supplementary Figure 6

Supplementary Figure 7

Supplementary Table 1

Supplementary Table 2

Supplementary Figure Legends
