# Supplementary figures and images for: USP42 drives nuclear speckle mRNA splicing via directing dynamic phase separation to promote tumorigenesis
Source: Cell Death Differ. 2021 Mar 17;28(8):2482–98. doi: 10.1038/s41418-021-00763-6 (PMC8329168; doi:10.1038/s41418-021-00763-6)

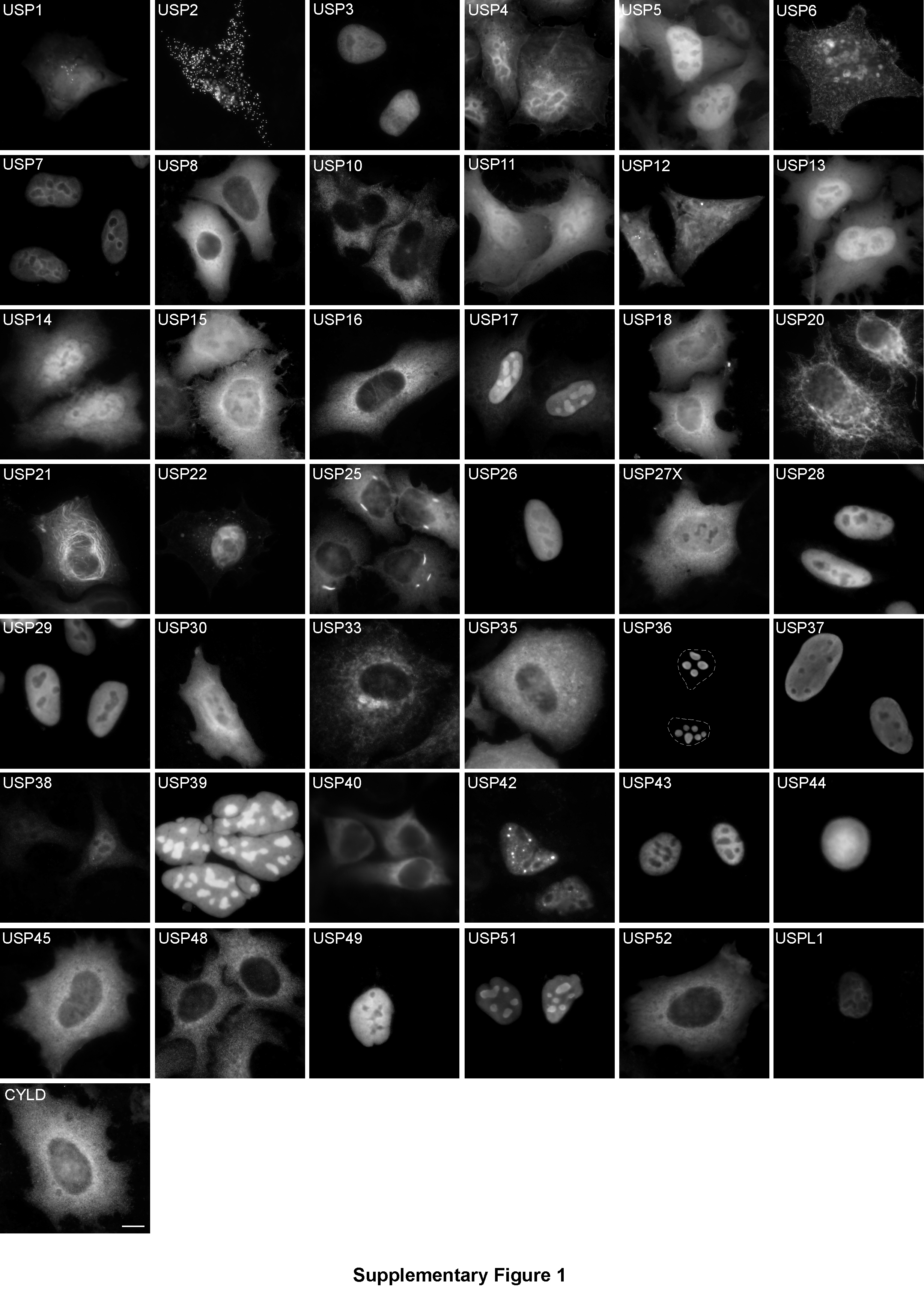

Supplement: Supplementary file 1 — Supplementary Figure 1 [file 41418_2021_763_MOESM1_ESM.tif]

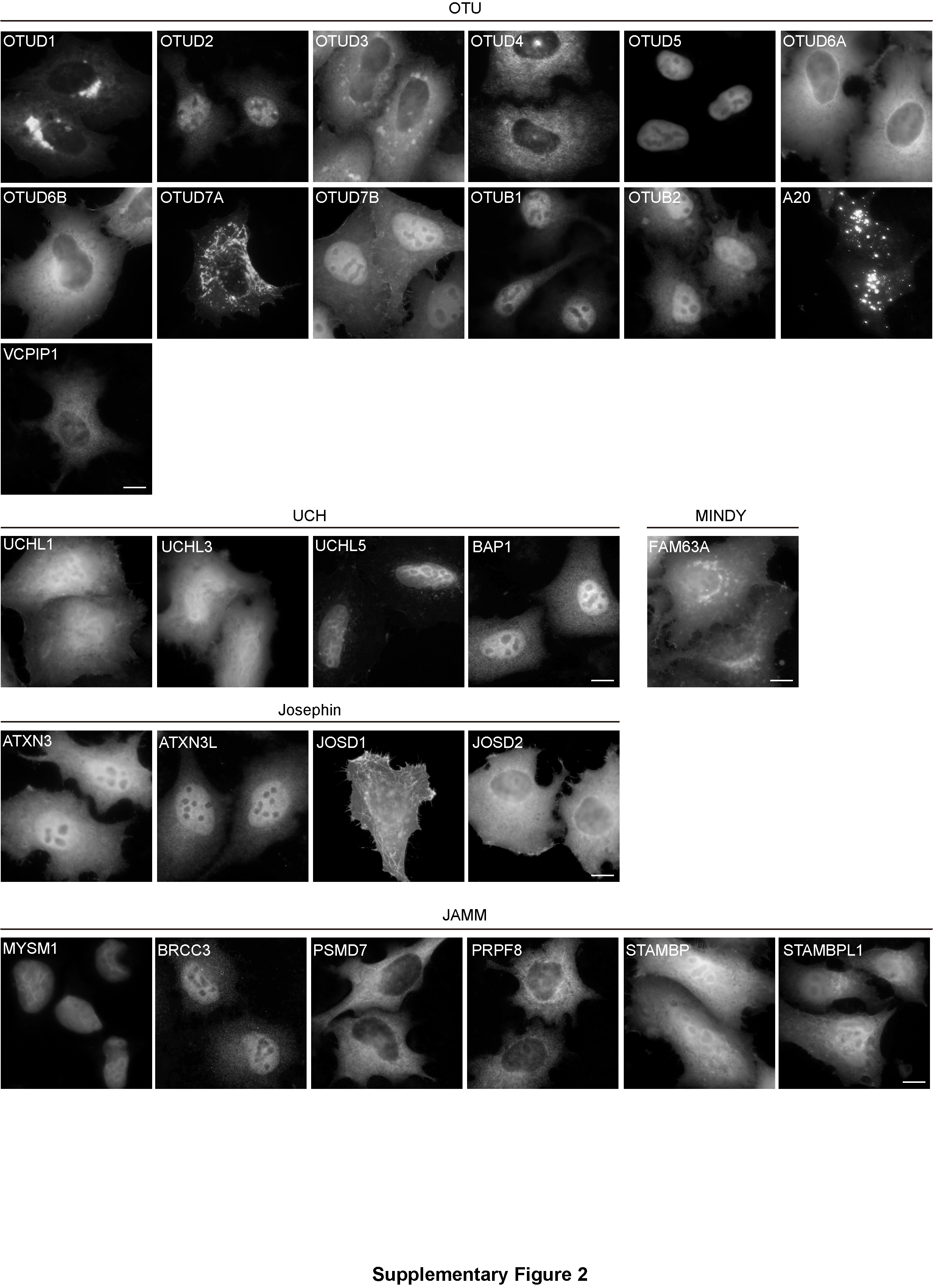

Supplement: Supplementary file 2 — Supplementary Figure 2 [file 41418_2021_763_MOESM2_ESM.tif]

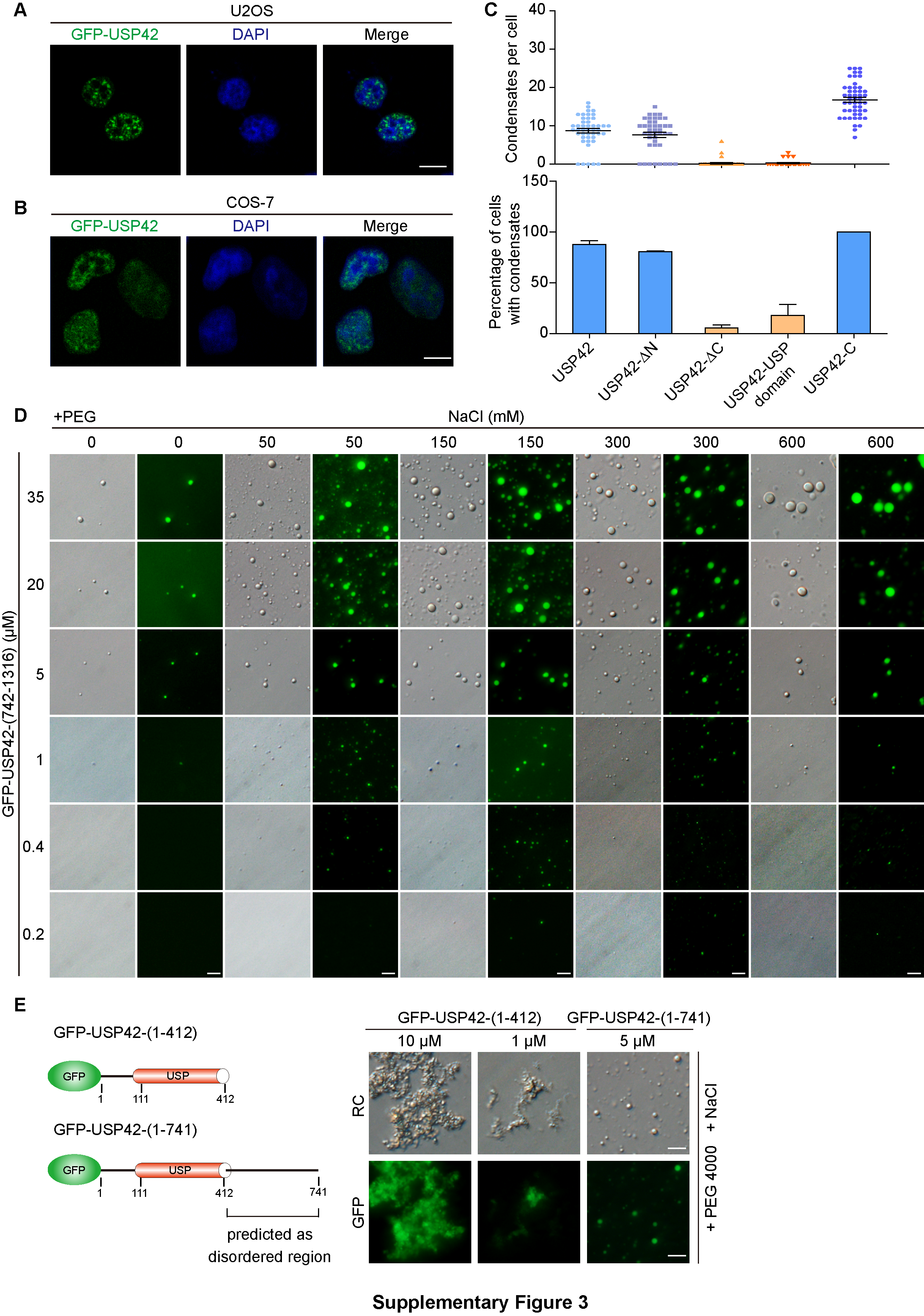

Supplement: Supplementary file 3 — Supplementary Figure 3 [file 41418_2021_763_MOESM3_ESM.tif]

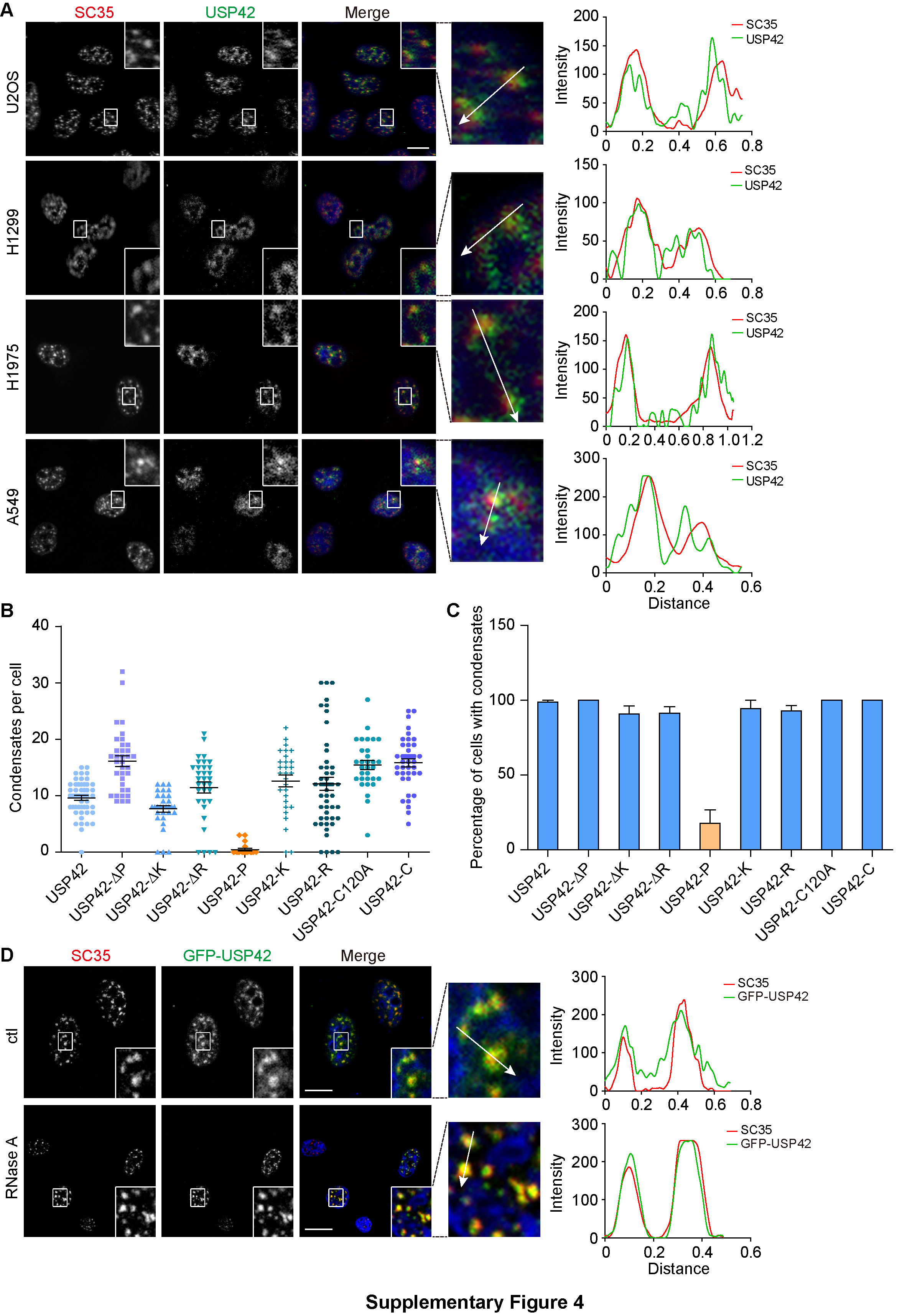

Supplement: Supplementary file 4 — Supplementary Figure 4 [file 41418_2021_763_MOESM4_ESM.tif]

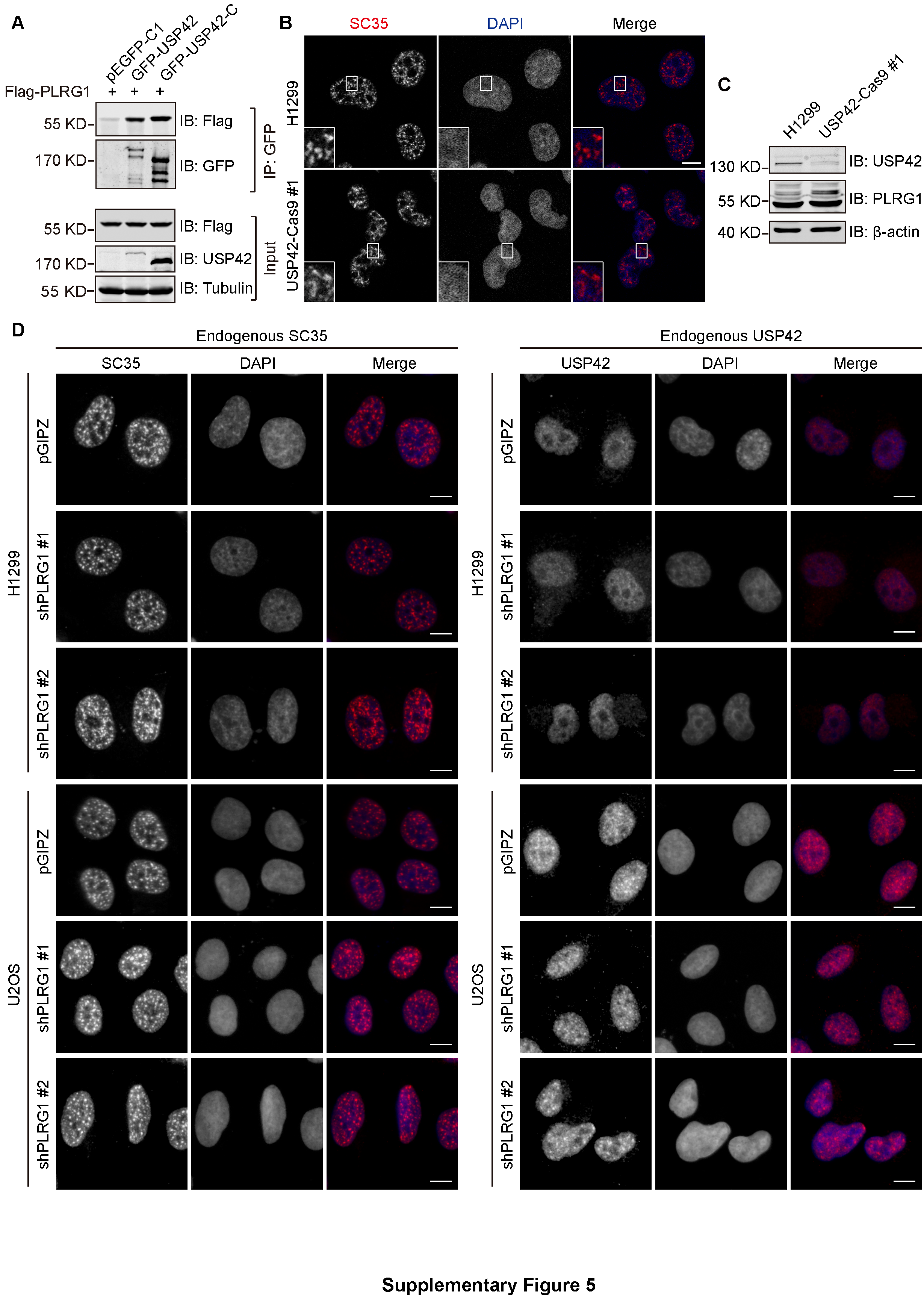

Supplement: Supplementary file 5 — Supplementary Figure 5 [file 41418_2021_763_MOESM5_ESM.tif]

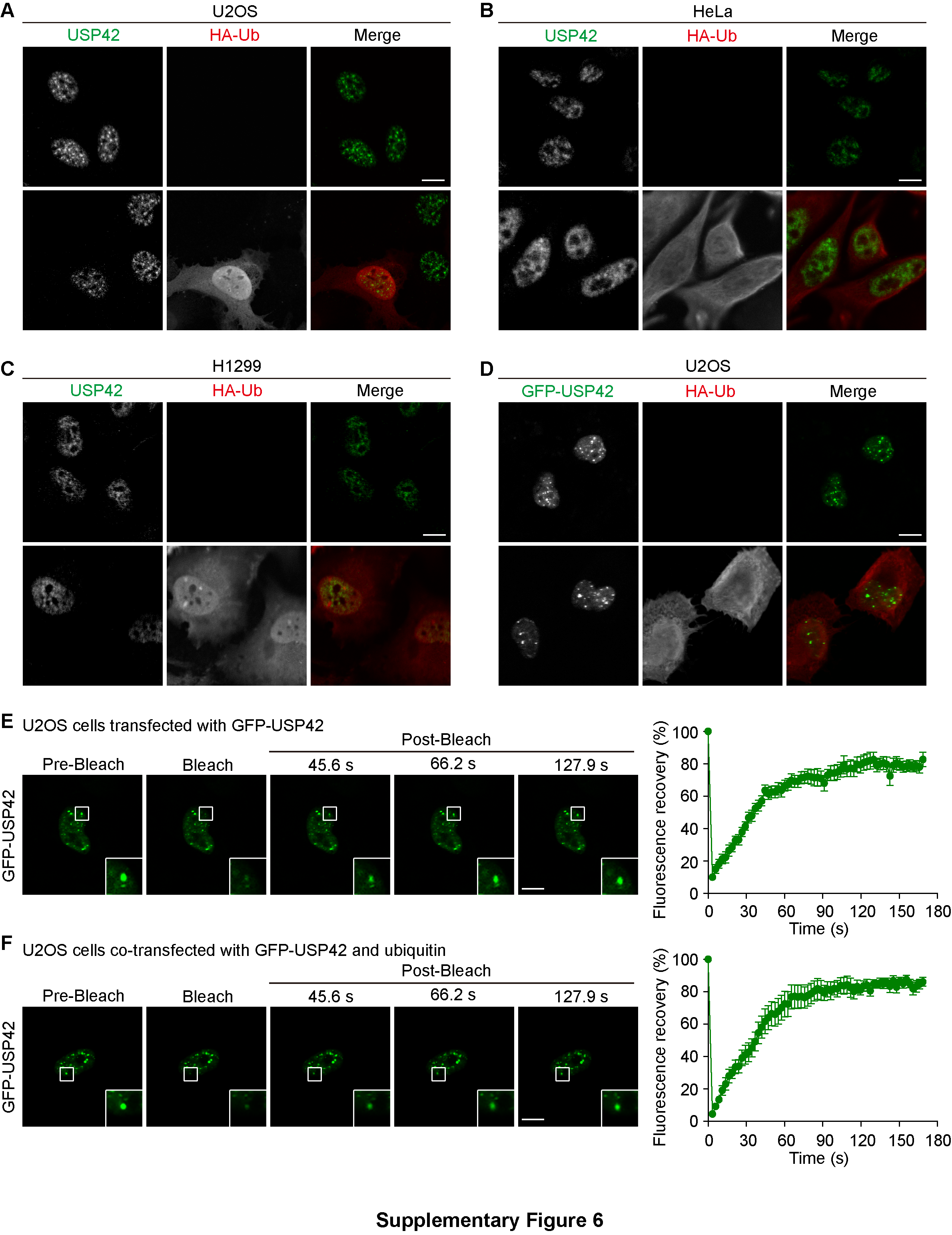

Supplement: Supplementary file 6 — Supplementary Figure 6 [file 41418_2021_763_MOESM6_ESM.tif]

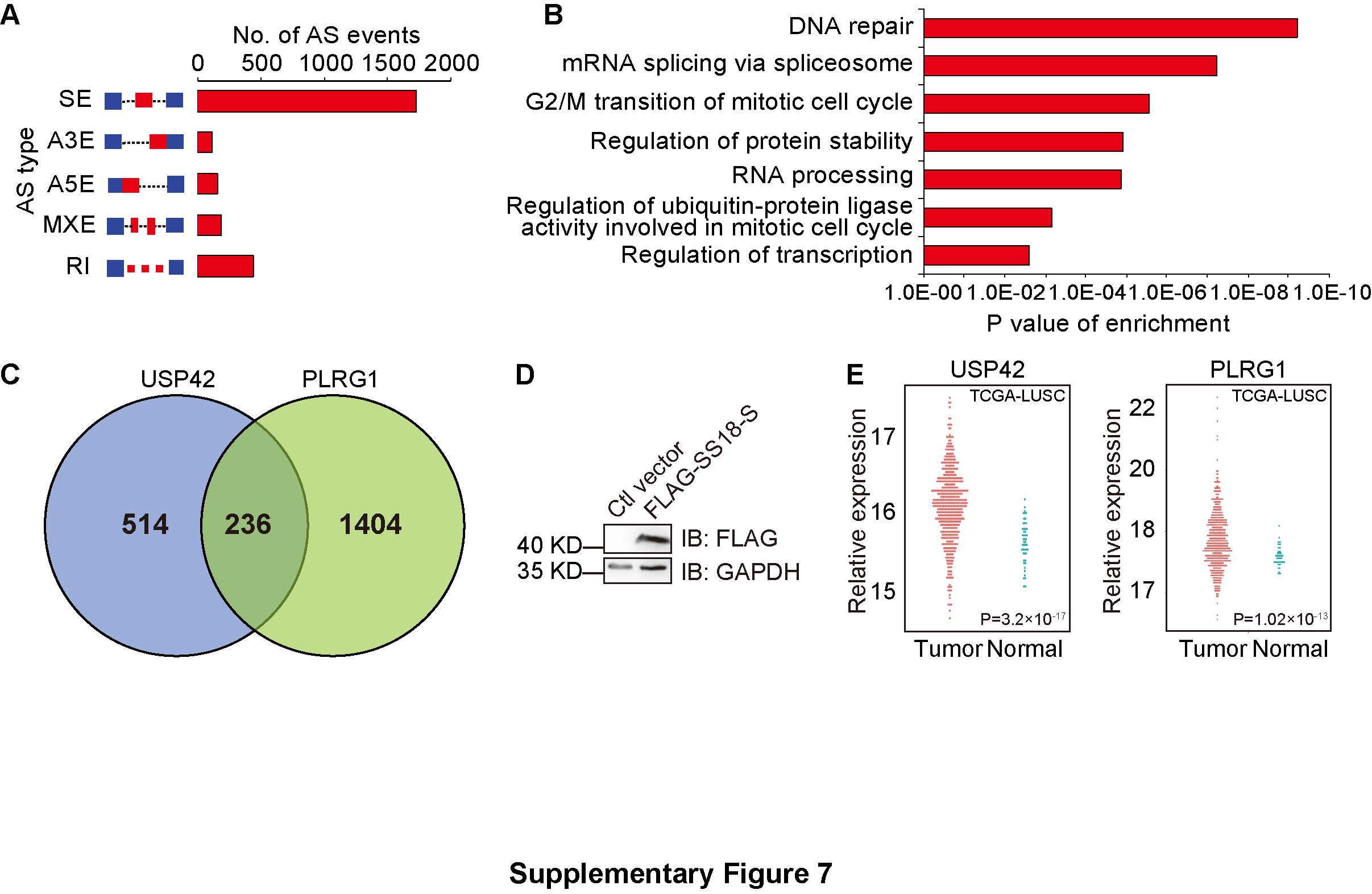

Supplement: Supplementary file 7 — Supplementary Figure 7 [file 41418_2021_763_MOESM7_ESM.tif]
