# Supplementary material for: USP42 drives nuclear speckle mRNA splicing via directing dynamic phase separation to promote tumorigenesis
Source: Cell Death Differ. 2021 Mar 17;28(8):2482–98. doi: 10.1038/s41418-021-00763-6 (PMC8329168; doi:10.1038/s41418-021-00763-6)
Supplement: Supplementary file 8 — Supplementary Table 1 [file 41418_2021_763_MOESM8_ESM.docx]

Supplementary Table 1. DUB expression constructs used in this study.

| **DUB** | **TAG** | **Reference** |
| --- | --- | --- |
| **USP** | | |
| USP1 | GFP | This study |
| USP2 | GFP | This study |
| USP3 | Flag-HA | Sowa et al. 2009 |
| USP4 | GFP | This study |
| USP5 | GFP | This study |
| USP6 | GFP | This study |
| USP7 | GFP | This study |
| USP8 | GFP | This study |
| USP10 | Flag-HA | Sowa et al. 2009 |
| USP11 | GFP | This study |
| USP12 | GFP | This study |
| USP13 | GFP | This study |
| USP14 | Flag-HA | Sowa et al. 2009 |
| USP15 | GFP | This study |
| USP16 | Flag-HA | Sowa et al. 2009 |
| USP17 | GFP | This study |
| USP18 | GFP | This study |
| USP20 | Flag-HA | Sowa et al. 2009 |
| USP21 | GFP | This study |
| USP22 | GFP | This study |
| USP25 | Flag-HA | Sowa et al. 2009 |
| USP26 | GFP | This study |
| USP27X | GFP | This study |
| USP28 | GFP | This study |
| USP29 | GFP | This study |
| USP30 | GFP | This study |
| USP33 | GFP | This study |
| USP35 | GFP | This study |
| USP36 | Flag-HA | Sowa et al. 2009 |
| USP37 | Flag-HA | Sowa et al. 2009 |
| USP38 | Flag-HA | Sowa et al. 2009 |
| USP39 | GFP | This study |
| USP40 | GFP | This study |
| USP42 | GFP | This study |
| USP43 | Flag-HA | Sowa et al. 2009 |
| USP44 | GFP | This study |
| USP45 | Flag-HA | Sowa et al. 2009 |
| USP48 | Flag-HA | Sowa et al. 2009 |
| USP49 | GFP | This study |
| USP51 | GFP | This study |
| USP52 | GFP | This study |
| USPL1 | GFP | This study |
| CYLD | Flag-HA | Sowa et al. 2009 |
| **OTU** | | |
| OTUD1 | GFP | This study |
| OTUD2 | Flag-HA | Sowa et al. 2009 |
| OTUD3 | GFP | This study |
| OTUD4 | GFP | This study |
| OTUD5 | GFP | This study |
| OTUD6A | GFP | This study |
| OTUD6B | GFP | This study |
| OTUD7A | GFP | This study |
| OTUD7B | GFP | This study |
| OTUB1 | Flag-HA | Sowa et al. 2009 |
| OTUB2 | Flag-HA | Sowa et al. 2009 |
| A20 | GFP | This study |
| VCPIP1 | Flag-HA | Sowa et al. 2009 |
| **UCH** | | |
| UCHL1 | GFP | This study |
| UCHL3 | GFP | This study |
| UCHL5 | GFP | This study |
| BAP1 | Flag-HA | Sowa et al. 2009 |
| **MINDY** | | |
| FAM63A | GFP | This study |
| **Josephin** | | |
| ATXN3 | Flag-HA | Sowa et al. 2009 |
| ATXN3L | GFP | This study |
| JOSD1 | Flag-HA | Sowa et al. 2009 |
| JOSD2 | Flag-HA | Sowa et al. 2009 |
| **JAMM** | | |
| MYSM1 | GFP | This study |
| BRCC3 | Flag-HA | Sowa et al. 2009 |
| PSMD7 | Flag-HA | Sowa et al. 2009 |
| PRPF8 | GFP | This study |
| STAMBP | GFP | This study |
| STAMBPL1 | GFP | This study |
