# Supplementary material for: USP42 drives nuclear speckle mRNA splicing via directing dynamic phase separation to promote tumorigenesis
Source: Cell Death Differ. 2021 Mar 17;28(8):2482–98. doi: 10.1038/s41418-021-00763-6 (PMC8329168; doi:10.1038/s41418-021-00763-6)
Supplement: Supplementary file 9 — Supplementary Table 2 [file 41418_2021_763_MOESM9_ESM.docx]

Supplementary Table 2. Primers used in cloning and RT-PCR experiments. All sequences are given in the order of 5’ to 3’.

| Primers | Sense | Anti-sense |
| --- | --- | --- |
| USP42 | GCTCTCGAGCTATGACCATAGTTGACAAAGCTT | CCAGGTACCTCAATCACCCTGGCCATACTCAA |
| USP42-∆N | GCTCTCGAGCTGCTGGGCTCCAGAATTTGG | CCAGGTACCTCAATCACCCTGGCCATACTCAA |
| USP42-∆C | GCTCTCGAGCTATGACCATAGTTGACAAAGCTT | CCTGGTACCTCAGGACCTGATATAAAAGAGCAC |
| USP42-USP domain | GCTCTCGAGCTGCTGGGCTCCAGAATTTGG | CCTGGTACCTCAGGACCTGATATAAAAGAGCAC |
| USP42-C | GCTCTCGAGCTCATGATGTGAAAAATGGAGGTG | CCAGGTACCTCAATCACCCTGGCCATACTCAA |
| USP42-(742-1316) | CCGAATTCCCTCCCGAGGACCGCGACGC | TGCTCGAGTCAATCACCCTGGCCATACTC |
| USP42-∆P | GAGAGGGGCGCGAAGGAGAAAATCGGCAG | CTCCTTCGCGCCCCTCTCTGCTCCAGG |
| USP42-∆K | GTCATGTTGAATCAGAGGACTTTGTTAAAGATTC | AAGTCCTCTGATTCAACATGACTGTCACTGTTC |
| USP42-∆R | GGCAGCCTCGCAGGCGCGCCCCACG | GCGCCTGCGAGGCTGCCGATTTTCTCC |
| USP42-C120A | CAGAATTTGGGCAATACCGCTTTTGCCAATGCAGCACTG | CAGTGCTGCATTGGCAAAAGCGGTATTGCCCAAATTCTG |
| GFP-PLRG1 | GCATGGACGAGCTGTACAAGATGGTCGAGGAGGTACAGAA | TTCGCGGCCGCTTAAAATCTCTTTCTCTTGATAATTTCTG |
| ZNF207 | AGCCTCTTTTCCCCAGTGCT | GCAGGGAATGTAGGCTTTGG |
| SS18 | TTACGGGGACCAATACAGTC | GCTGCTGCTGGGGTGGATAT |
| PSAP | GCAAGAACTATATCAGCCAG | GGGCATCTCTTTCACCTCAT |
| NF2 | TGAAAGAGAGGGAGACAGCT | TAGCAGGAGAAGTGGCAGGT |
| LATS1 | TTGGGACGCATCATAAAGCC | CGTCGAGGATCTTGGTAACT |
| FOXM1 | ACCGCTACTTGACATTGGAC | GGGAGTTCGGTTTTGATGGT |
| ATP5SL | GACATCAACTACGAGGGCCT | GCAGCATCTCCTCCACCAAT |
| CEP57 | AGAAGAGCACGAGAACCTAG | GGAGATGAAGAATGCCGAAC |
| SDCCAG8 | GGGCGAAACCTCCAAAAGAC | GGGATTCCTCAATTTCACAC |
