# Supplementary material for: USP42 drives nuclear speckle mRNA splicing via directing dynamic phase separation to promote tumorigenesis
Source: Cell Death Differ. 2021 Mar 17;28(8):2482–98. doi: 10.1038/s41418-021-00763-6 (PMC8329168; doi:10.1038/s41418-021-00763-6)
Supplement: Supplementary file 10 — Supplementary Figure Legends [file 41418_2021_763_MOESM10_ESM.docx]

Supplementary Figure 1. Intracellular localization of 43 deubiquitylases from the USP subfamily. Forty-three USPs tagged with GFP or Flag described in Supplementary Table 1 were transiently expressed in HeLa cells for 48 h, and fluorescence assays were conducted to visualize intracellular localizations. Scale bar = 10 μm.

Supplementary Figure 2. Intracellular localization of 28 deubiquitylases from the OTU, UCH, MINDY, Josephin, and JAMM subfamilies. Twenty-eight GFP- or Flag-tagged deubiquitylases described in Supplementary Table 1 were exogenously expressed in HeLa cells for 48 h, followed by fluorescence assays to detect intracellular distributions of these deubiquitylases. Scale bar = 10 μm.

Supplementary Figure 3. Characterization of the phase separation properties of USP42. A and B, Cultured U2OS and COS-7 cells were transfected with constructs expressing GFP-tagged USP42 wild-type before fluorescence analysis to examine its intracellular distribution. Nucleus was stained with DAPI. Scale bar = 10 μm. C, Quantification data of condensates formed by GFP-tagged USP42 variants showing condensates per cell and the percentage of cells with condensates in U2OS cells expressing various variants as indicated in Figure 2C. Error bars represent standard error of the mean. D, Phase separation of bacterially purified GFP-tagged USP42-(742-1316) was assessed using *in vitro* assays with indicated concentrations (0.2, 0.4, 1, 5, 20, 35 μM). The crowding agent PEG 4000 was added to each sample at a final concentration of 25% (w/v). The concentration for NaCl was titrated from 0 to 600 mM as indicated. Representative relief contrast (RC) and fluorescence images are shown. Scale bar = 5 μm. E, Representative RC and fluorescence images showing phase separation properties of indicated GFP-tagged USP42-(1-412) and USP42-(1-741). Schematic diagram shows domain architectures. Scale bar = 5 μm.

Supplementary Figure 4. Characterization of nuclear speckle localization of USP42. A, The colocalization of endogenous USP42 with SC35 in U2OS, H1299, H1975, and A549 cells by immunofluorescence and confocal assays. Representative micrographs were shown with magnified insets. The graphs on the right show the quantification of fluorescence intensities for SC35 (red) and USP42 (green). Nucleus were stained with DAPI. Scale bar = 10 μm. B and C, Quantification of condensates formed by GFP-tagged USP42 variants in U2OS cells from 3 independent experiments as described in Figures 3 and 4. Error bars represent standard error of the mean. D, U2OS cells transfected with GFP-USP42 construct were treated with or without RNase A (100 µg/ml) following permeabilization with Triton X-100 (0.05%) before standard immunofluorescence analysis. Representative confocal micrographs with magnified insets are shown to illustrate colocalization of GFP-USP42 with SC35. Nucleus was stained with DAPI. Scale bar = 10 μm. Graphs on the right show quantification of fluorescence intensity.

Supplementary Figure 5. USP42 associates with PLRG1 and regulates nuclear speckles. A, GFP-tagged wild-type and C-terminus of USP42 were expressed in HEK293T cells along with Flag-tagged PLRG1 for 48 h and lysed. GFP-tagged USP42 proteins were immunoprecipitated using anti-GFP antibodies and analyzed by immunoblotting with indicated antibodies together with cell lysate samples (input). Tubulin was detected to confirm equal loading. B, Immunofluorescence assays to detect nuclear speckles marked with SC35 staining in parental and USP42-Cas9 H1299 cells. Representative confocal images are shown with magnified insets to demonstrate SC35-labeled speckles. Nucleus was stained with DAPI. Scale bar = 10 μm. C, PLRG1 levels in parental and USP42-Cas9 H1299 cells were examined by immunoblotting with indicated antibodies. β-actin blot shows equal loading. D, Immunofluorescence assays showing the distribution of endogenous SC35 and USP42 in indicated cells with PLRG1 depletion. Nucleus were stained with DAPI. Scale bar = 10 μm.

Supplementary Figure 6. Characterization of USP42 condensates with ubiquitin overexpression. A-C, Immunofluorescence assays showing the distribution of endogenous USP42 in U2OS, HeLa, and H1299 cells with or without HA-ubiquitin (HA-Ub) overexpression. Representative confocal micrographs were presented. Scale bar = 10 μm. D, U2OS cells were transfected with constructs expressing GFP-tagged USP42 or together with HA-tagged ubiquitin plasmid. Representative confocal micrographs show the distribution of GFP-USP42. Scale bar = 10 μm. E and F, FRAP analyses of GFP-tagged USP42 droplets in U2OS cells with or without ubiquitin overexpression as indicated. Representative time-lapse images with magnified insets were presented to show FRAP recovery of punctae formed by GFP-USP42. The graphs on the right show quantification of relative fluorescence intensities pre- and post-bleach (n = 3). Scale bar = 10 μm.

Supplementary Figure 7. Analysis of alternative splicing regulated by PLRG1. A, Quantification of the different AS events affected by PLRG1 knockdown. B, Gene ontology analysis of PLRG1-regulated AS targets. Fisher exact P values were plotted for each enriched functional category. C, The overlapped AS events regulated by USP42 and PLRG1 were demonstrated. D, The protein levels of SS18-S in H1299 cells stably transfected with control or SS18-S expression vectors were measured by immunoblotting using indicated antibodies. E, The mRNA levels of USP42 and PLRG1 in lung squamous carcinoma and normal tissues were analyzed using TCGA dataset, with P values calculated by unpaired t-test.
